# Editorial: Natural products for neuroprotection and neuroregeneration

**DOI:** 10.3389/fphar.2023.1209297

**Published:** 2023-05-17

**Authors:** Kah Hui Wong, Lee Wei Lim, Nur Shahirah Mohd Hisam, Muhamad Noor Alfarizal Kamarudin, Hariprasath Lakshmanan

**Affiliations:** ^1^ Department of Anatomy, Faculty of Medicine, Universiti Malaya, Kuala Lumpur, Malaysia; ^2^ School of Biomedical Sciences, Li Ka Shing Faculty of Medicine, The University of Hong Kong, Hong Kong, Hong Kong SAR, China; ^3^ Jeffrey Cheah School of Medicine and Health Sciences, Monash University Malaysia, Bandar Sunway, Selangor, Malaysia; ^4^ Division of Biochemistry, School of Life Sciences, JSS Academy of Higher Education and Research, Mysuru, Karnataka, India

**Keywords:** blood-brain barrier, cognitive function, ER stress, motor deficit, mitochondrial function, neurodegeneration, neuroinflammation

Neuroregeneration is a fairly new concept encompassing neurogenesis, neuroplasticity and neurorestoration. Nevertheless, it is a controversial Research Topic in the field of neuroscience due to limitations of study methods hampering neurogenesis related research in adult humans. Further, neuroregeneration exceeds the concept of neurogenesis that also contitutes endogenous neuroprotection leading to neuroplasticity and neurorestoration (Enciu et al., 2011; Muresanu et al., 2012; Huang and Chen, 2015). The past decade has witnessed an intense interest in natural products that offer health-promoting effects on neurodegenerative diseases through neuroprotection and/or neuroregeneration (John et al., 2013; Phan et al., 2014; Samberkar et al., 2015; Pang et al., 2018; Chong et al., 2020; 2021; Lew et al., 2020; Subermaniam et al., 2020; Phang et al., 2021; Choy et al., 2022; Wong et al., 2022).

This Research Topic served as a networking platform to gather scientists in the field of ethnopharmacological research to share cutting-edge research and reviews related to therapeutic efficacy of natural products for the treatment of neurodegenerative diseases (Figure 1). The main objective of this Research Topic was to address the key questions with regards to molecular mechanisms in the attenuation of programmed cell death and neuroinflammation, improvement of microcirculation in the brain, and restoration of synaptic failure and altered neurogenesis, justifying their therapeutic roles.

**FIGURE 1:**
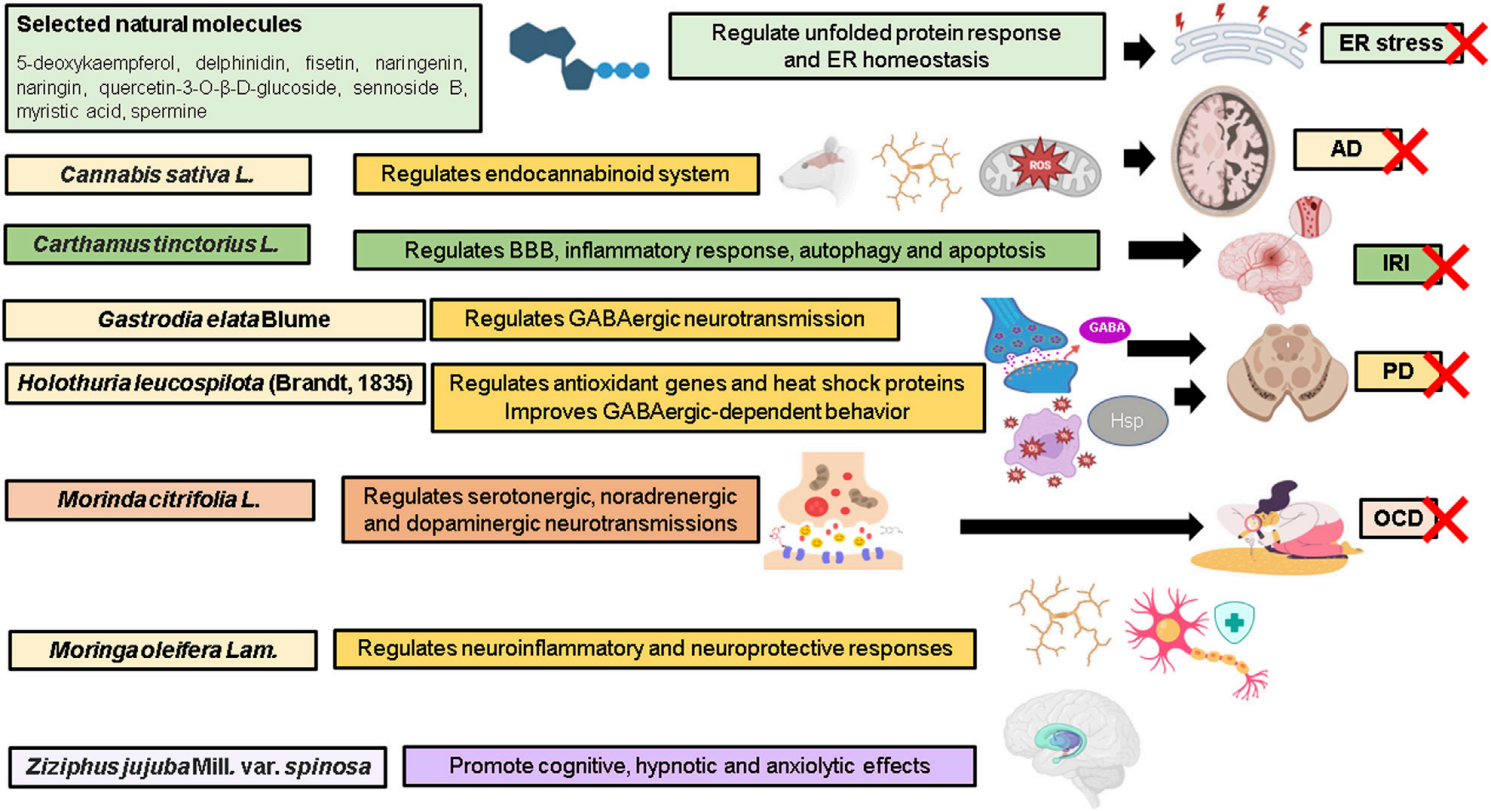
Highlights of the Research Topic. AD, Alzheimer’s disease; BBB, blood brain barrier; ER, endoplasmic reticulum; GABA, gamma-aminobutyric acid; Hsp heat-shock protein; IRI, ischemia-reperfusion injury; OCD, obsessive compulsive disorder; PD, Parkinson’s disease; ROS, reactive oxygen species; X, attenuate. Illustration created with BioRender.com.

da Silva et al. investigated the protective roles of 134 natural molecules against endoplasmic reticulum (ER) stress in *in vitro* models of MRC-5 fibroblasts and SH-SY5Y cells exposed to thapsigargin. Of these, 5-deoxykaempferol, delphinidin, fisetin, naringenin, naringin, quercetin-3-O-β-D-glucoside, sennoside B, myristic acid, and spermine appear to be potential candidates in maintaining ER homeostasis. The major cellular mechanisms include attenuation of protein aggregation and calcium overload, and activation of unfolded protein response.

For the past 3 decades, *Cannabis sativa* L. (cannabis) has been found to regulate cognitive and emotional processing. Cannabinoids represent the most studied group of compounds, mainly due to their pharmaceutical effects in humans such as psychotropic activities. Kamaruzzaman et al. presented a systematic review and meta-analysis revealing the modulatory roles of cannabinoids on endocannabinoid system in rodent models of Alzheimer’s disease, leading to restoration of cognitive behavior. A total of 26 studies were included and systematically evaluated. Profound alterations were observed in the pattern of expression of cannabinoid receptor type II (CB_2_) receptors and fatty acid amide hydrolase (FAAH) in the brain. These changes are linked to the inflammatory response, suggesting a crucial role for the endocannabinoid system in glial activation. The process is characterized by transformation of glial cell phenotype, upregulation and downregulation of anti-inflammatory cytokines and pro-inflammatory cytokines, respectively, glial autophagy for the clearance of aggregates, inhibition of ROS/RNS generation and lipid peroxidation, as well as modulation of synaptic plasticity.

Yu et al. presented a review on hydroxysafflor yellow A (HSYA), a major compound derived from *Carthamus tinctorius* L. (safflower). A total of 14 *in vitro* and 17 *in vivo* studies provided evidence of clinical promise, indicating saffron and HSYA are indeed safe for consumption to improve diverse clinical outcomes and therefore can be considered effective for the treatment of ischemia stroke and reperfusion injury. HSYA has been reported to inhibit excitotoxicity, oxidative stress, preserve blood-brain barrier, and regulate key pathophysiological processes such as inflammation, autophagy and apoptosis.

Lu et al. presented a review on the neuroprotective effects of bioactive components and extracts of Gastrodia elata Blume (Tianma) rhizome in preclinical models of Parkinson’s disease. Of 81 bioactive compounds isolated and identified, gastrodin, vanillyl alcohol, vanillin, vanillic acid, and anisalcohol have been observed to confer neuroprotective activities targeting aggregation of α-synuclein, vulnerability of dopaminergic neurons in the substantia nigra and neuroinflammation. These compounds improved motor and cognitive functions through Nrf2-mediated antioxidant defense system regulating a battery of antioxidant and cellular protective genes, restoration of mitochondrial function, attenuation of microglial activation and oxidative stress, downregulation of c-jun N-terminal kinase (JNK)-nuclear factor-κB (NF-κB) pathway and facilitation of GABAergic neurotransmission. Additionally, Sanguanphun et al. demonstrated the neuroprotective effects of decanoic acid isolated from Holothuria leucospilota (Brandt, 1835) or black sea cucumber (HLEA-P1) in the alleviation of Parkinsonism in an *in vivo* model of *Caenorhabditis elegans* exposed to neurotoxin 6-hydroxydopamine (6-OHDA). The HLEA-P1 attenuated oxidative stress leading to suppression of aggregation of α-synuclein and intracellular deposition of lipid droplets, activated insulin/insulin-like growth factor (IGF-1) signaling (IIS) pathway and upregulated antioxidant genes and heat-shock proteins, contributing to improved GABAergic-dependent behavior.

Jeyabalan et al. demonstrated the neuroprotective potential of a standardized fruit extract of *Morinda citrifolia* L., commonly known as noni, against obsessive-compulsive disorder (OCD)-like behavioral traits in a mouse model. Oral administration of the extract suppressed nestlet shredding and marble burying without affecting the locomotor function. Importantly, this study suggests that the attenuation of OCD-like behavior has been observed to be associated with amelioration of biogenic amines and elevation of serotonin levels and regulation of serotonergic, noradrenergic and dopaminergic neurotransmission.

Azlan et al. presented a review on Moringa oleifera Lam., popularly known as a drumstick tree or tree of life. The herbal medicine possesses neuroprotective and anti-neuroinflammatory effects by modulating the levels of NF-κB, cytokines, TNF-α, IL-1, IL-6, and nitric oxide (NO), leading to the suppression of inflammatory reaction. The therapeutic effects are associated with the abundance of phytochemicals rich in antioxidant and anti-inflammatory properties, namely, phenolic acids (gallic, chlorogenic, ferulic, and caffeic acids), flavonoids (kaempferol, myricetin, (−)- epicatechin, quercetin, isoquercitrin and astragalin), glucosinolates (GLSs) and isothiocyanates (ITCs and moringin). However, data on their pharmacokinetic properties in preclinical models are lacking. This review also discusses toxicity-related Research Topic and major safety concerns. Accumulating evidence shows that M. oleifera extracts and compounds are acceptably safe.

Kuang et al. presented a mini review on spinosin, a C-glycoside flavonoid isolated from the seeds of Ziziphus jujuba Mill. var. spinosa (red date) and demonstrated evidence in supporting the use of the compound for cognitive function, hypnosis and anxiolytic effects in preclinical models. However, there is a lack of in-depth molecular mechanisms, pharmacokinetics parameters, information content of nuclear magnetic resonance (NMR) spectra, toxicity assessment and network pharmacology to draw definitive conclusions on the effectiveness of spinosin.

We sincerely appreciate the insightful and constructive suggestions from the reviewers which helped us in improving the quality of the manuscripts.

## References

[B1] Chong P. S. Khairuddin S. Tse A. C. K. Hiew L. F. Lau C. L. Tipoe G. L. (2020). *Hericium erinaceus* potentially rescues behavioural motor deficits through ERK-CREB-PSD95 neuroprotective mechanisms in rat model of 3-acetylpyridine-induced cerebellar ataxia. Sci. Rep. 10, 14945. 10.1038/s41598-020-71966-z 32913245 PMC7483741

[B2] Chong P. S. Poon C. H. Roy J. Tsui K. C. Lew S. Y. Phang M. W. L. (2021). Neurogenesis-dependent antidepressant-like activity of *Hericium erinaceus* in an animal model of depression. Chin. Med. 16, 132. 10.1186/s13020-021-00546-8 34876186 PMC8650354

[B3] Choy K. W. Wong K. H. Abas R. Haron M. H. Das S. Teoh S. L. (2022). Natural product-based nanomedicine: Recent advances and issues for the treatment of Alzheimer's disease. Curr. Neuropharmacol. 20 (8), 1498–1518. 10.2174/1570159X20666211217163540 34923947 PMC9881085

[B4] Enciu A. M. Nicolescu M. I. Manole C. G. Mureşanu D. F. Popescu L. M. Popescu B. O. (2011). Neuroregeneration in neurodegenerative disorders. BMC Neurol. 11, 75. 10.1186/1471-2377-11-75 21699711 PMC3146817

[B5] Huang H. Chen L. Rieffel J. Lovell J. F. (2015). Emerging applications of porphyrins in photomedicine. J. Neurorestoratology 3, 23–30. 10.3389/fphy.2015.00023 PMC5445930 28553633

[B6] John P. A. Wong K.-H. Naidu M. Sabaratnam V. David P. (2013). Combination effects of curcumin and aqueous extract of *Lignosus rhinocerotis* mycelium on neurite outgrowth stimulation activity in PC-12 cells. Nat. Prod. Commun. 8 (6), 1934578X1300800. 10.1177/1934578X1300800608

[B7] Lew S. Y. Lim S. H. Lim L. W. Wong K. H. (2020). Neuroprotective effects of *Hericium erinaceus* (bull: Fr) pers against high-dose corticosterone-induced oxidative stress in PC-12 cells. BMC Complement. Med. Ther. 20, 340. 10.1186/s12906-020-03132-x 33176761 PMC7656699

[B8] Muresanu D. F. Buzoianu A. Florian S. I. von Wild T. (2012). Towards a roadmap in brain protection and recovery. J. Cell. Mol. Med. 16 (12), 2861–2871. 10.1111/j.1582-4934.2012.01605.x 22863521 PMC4393716

[B9] Pang J. R. Goh V. M. J. Tan C. Y. Phang S. M. Wong K. H. Yow Y. Y. (2018). Neuritogenic and *in vitro* antioxidant activities of Malaysian *Gracilaria manilaensis* yamamoto & trono. J. Appl. Phycol. 30, 3253–3260. 10.1007/s10811-018-1438-x

[B10] Phan C. W. David P. Tan Y. S. Naidu M. Wong K. H. Kuppusamy U. R. (2014). Intrastrain comparison of the chemical composition and antioxidant activity of an edible mushroom, *Pleurotus giganteus,* and its potent neuritogenic properties. Sci. World J. 2014, 378651. 10.1155/2014/378651 PMC4121195 25121118

[B11] Phang M. W. L. Lew S. Y. Chung I. Lim K.-S. Lim L. W. Wong K. H. (2021). Therapeutic roles of natural remedies in combating hereditary ataxia: A systematic review. Chin. Med. 16, 15. 10.1186/s13020-020-00414-x 33509239 PMC7841890

[B12] Samberkar S. Gandhi S. Naidu M. Wong K.-H. Raman J. Sabaratnam V. (2015). Lion’s mane, *Hericium erinaceus* and Tiger milk, *Lignosus rhinocerotis* (higher basidiomycetes) medicinal mushrooms stimulate neurite outgrowth in dissociated cells of brain, spinal cord, and retina: An *in vitro* study. Int. J. Med. Mushrooms 17, 1047–1054. 10.1615/intjmedmushrooms.v17.i11.40 26853959

[B13] Subermaniam K. Yow Y. Y. Lim S. H. Koh O. H. Wong K. H. (2020). Malaysian macroalga *Padina australis* Hauck attenuates high dose corticosterone-mediated oxidative damage in PC12 cells mimicking the effects of depression. Saudi J. Biol. Sci. 27 (6), 1435–1445. 10.1016/j.sjbs.2020.04.042 32489279 PMC7254034

[B14] Wong K. H. Nam H. Y. Lew S. Y. Naidu M. David P. Kamalden T. A. (2022). Discovering the potential of natural antioxidants in age-related macular degeneration: A review. Pharmaceuticals 15 (1), 101. 10.3390/ph15010101 35056157 PMC8777838

